# The mediating effects of convergence of femininity-maternity and marital intimacy in the relationship between postpartum depression and quality of life in postpartum mothers: a cross-sectional study

**DOI:** 10.4069/whn.2024.12.17

**Published:** 2025-03-28

**Authors:** Ye Jin Hong, Hae Ok Kim

**Affiliations:** 1 Department of Nursing, Samsung Changwon Hospital, Sungkyunkwan University School of Medicine, Changwon, Korea; 2 College of Nursing, Kyungnam University, Changwon, Korea

**Keywords:** Postpartum, Postpartum depression, Quality of life, Spouse, Women

## Abstract

**Purpose:**

This study explored the mediating effect of convergence of maternity‐femininity and marital intimacy in the relationship between postpartum depression (PPD) and quality of life (QoL) in postpartum mothers.

**Methods:**

Women between 2-6 weeks after childbirth were recruited from three general hospitals in Changwon, Korea, and an online community in March 2023. Descriptive statistics, the t-test, one‐way analysis of variance, and Pearson correlation coefficients were computed. The mediating effects of convergence of maternity‐femininity and marital intimacy in the relationship between PPD and QoL were assessed using Hayes’ PROCESS Macro Model 4.

**Results:**

The mean QoL score was relatively low (17.77±3.83), while the mean PPD score was 12.06±6.79; 64.5% of participants scored ≥10, indicating PPD. The mean score for convergence of maternity‐femininity was 118.84±19.85. Marital intimacy was mid-point (55.95±10.40). Convergence of maternity‐femininity exhibited a partial mediating effect on the relationship between PPD and QoL, with 56.0% of the overall effect of PPD on QoL being indirect via this mediator.

**Conclusion:**

PPD may affect QoL both directly and indirectly through its impact on convergence of maternity‐femininity. Therefore, interventions should not only address PPD but also promote convergence of maternity‐femininity to enhance the QoL of postpartum mothers. For example, psychological counseling to support emotional stability, educational programs to integrate femininity and maternity, partner involvement workshops to encourage spousal support, linkage to community resources, and exercise and wellness programs to foster a positive body image may all be beneficial.

## Introduction

The low birth rate in Korean society has reached critical levels, with the fertility rate falling below one per woman—recorded at 0.98 in 2018, 0.81 in 2021, and 0.78 in 2022 [1]. As low fertility emerges as a significant social issue, a multidisciplinary approach is essential [2]. In particular, providing effective nursing services during pregnancy and childbirth is crucial for enhancing women’s quality of life (QoL) [3].

Postpartum mothers undergo rapid physical and emotional changes after childbirth [4]. They frequently experience various physical discomforts, including rapid hormonal fluctuations, breast engorgement from lactation, perineal pain, general postpartum pain, and bowel dysfunction [5]. These physical challenges are often accompanied by emotional strain, such as anxiety, tension, and an increased sense of responsibility, which can adversely affect overall QoL [6].

Postpartum depression (PPD) is a major factor that negatively impacts the QoL of postpartum mothers [7]. More than half of women (52.6%) experience some degree of depressive symptoms, and 10% to 15% may develop clinical PPD [8]. Because PPD can evolve into a chronic condition [9], timely and appropriate management is essential. Negative emotions and stress resulting from PPD undermine maternal self‐confidence, impair mother-infant interactions [10], and hinder adaptation to the maternal role [11]. Thus, preventive and interventional measures for PPD are crucial, as its adverse effects extend to both maternal role adaptation and newborn care.

Convergence of maternity‐femininity refers to the process by which postpartum mothers maintain their feminine identity while adapting to their maternal role, reflecting the modern woman’s desire for a fulfilling sense of motherhood [12]. Preserving a feminine identity without exclusively focusing on motherhood may enhance postpartum women’s QoL; therefore, it is necessary to examine the factors that influence this convergence. Despite its significance, research on convergence of maternity‐femininity in postpartum women remains limited, making it essential to determine whether—and to what extent—it affects QoL.

Maternal depression can strain spousal relationships, thereby negatively impacting marital intimacy [13]. Spousal support—including emotional comfort, encouragement, and collaborative problem-solving—is crucial for easing the transition into parenthood and reducing depressive symptoms [14]. Research indicates that higher levels of marital intimacy are associated with improved QoL in postpartum mothers [15]; greater intimacy correlates with lower levels of depression and anxiety, thereby enhancing overall well-being [16]. These findings underscore the critical role of spousal emotional and psychological support during postpartum recovery.

Furthermore, postpartum mothers with lower levels of PPD tend to adapt better to their maternal role [17], which contributes to improved QoL [18]. Conversely, PPD has been linked to strained spousal relationships and marital conflicts [13], although higher levels of marital intimacy may help mitigate these adverse effects [15].

While previous studies have explored these relationships, research on how convergence of maternity‐femininity and marital intimacy mediate the association between PPD and QoL remains limited. Therefore, this study aimed to assess postpartum mothers’ QoL, PPD, convergence of maternity‐femininity, and marital intimacy, as well as to examine the mediating effects of convergence of maternity‐femininity and marital intimacy on the PPD-QoL relationship. The objective was to provide foundational data for developing nursing interventions to improve postpartum mothers’ QoL.

## Methods

**Ethics statement:** This study was approved by the Institutional Review Board of Samsung Medical Center (No. SCMC 2022-12-006-002). Informed consent was obtained from the participants.

### Study design

This descriptive survey aimed to determine the relationships among QoL, PPD, convergence of maternity‐femininity, and marital intimacy in postpartum mothers, and to verify the mediating effects of convergence of maternity‐femininity and marital intimacy on the relationship between PPD and QoL. Data were collected using a structured self-report questionnaire from March 17 to 30, 2023. The study was described in accordance with the STROBE guidelines (https://www.strobe-statement.org/).

### Participants

Postpartum mothers between 2 and 6 weeks after childbirth were recruited from three general hospitals in Changwon, Korea, and an online community. To minimize selection bias, both hospital-based and online recruitment methods were employed, ensuring voluntary participation. The 2-6 weeks period was chosen because it is a critical time when maternity begins to form and stabilize—following the period of heavy dependency on postpartum support—making it an appropriate time to assess convergence of maternity‐femininity [12].

Inclusion criteria required mothers who had delivered a healthy newborn (born at 37–42 weeks gestation with a birth weight of at least 2,500 g), who could communicate in Korean, understood the study’s purpose, and provided informed consent. Mothers with complications during delivery or the postpartum period—such as preterm labor, gestational hypertension or diabetes mellitus, placental or uterine disorders, intrauterine growth restriction, or peripartum hemorrhage—were excluded. The required sample size was calculated using G*Power 3.1.9.7 based on predictor variables from previous studies investigating the relationship between PPD and QoL in postpartum mothers [4,19-21]. Previous research [19] indicated that at least 184 participants were needed for the multiple regression analysis, based on a significance level of 0.05, a medium effect size of 0.15, a statistical power of 0.95, and 13 predictors. To account for an estimated 20% dropout rate, 230 questionnaires were distributed; data from 197 participants (85.7%) were included in the final analysis.

### Study tools

#### Postpartum quality of life

The Maternal Postpartum Quality of Life (MAPP-QOL) instrument, developed by Hill et al. [22] based on Ferrans and Powers Quality of Life Index [23] and translated by Choi et al. [4], was used with permission.

The MAPP-QOL consists of 40 items divided into five subdomains: health and functioning (eight items), relation/family-friends (10 items), relation/spouse-partner (six items), psychological (eight items), and socioeconomic (eight items). Each item is rated on a 6-point Likert scale; satisfaction items range from 1 (“very dissatisfied”) to 6 (“very satisfied”), and importance items range from 1 (“not important at all”) to 6 (“very important”).

To calculate the MAPP-QOL score, satisfaction scores are first centered by subtracting 3.5 from each satisfaction response. The adjusted satisfaction score is then multiplied by its corresponding importance score to produce a weighted score for each item. The weighted scores are summed, and the total is divided by the number of items answered to account for missing data. Finally, 15 is added to eliminate negative values, resulting in a final QoL score ranging from 0 to 30, with higher scores indicating better QoL. At its development, Ferrans and Powers Quality of Life Index [23] demonstrated an overall Cronbach’s α of .96, with subdomain values of .91 (psychological), .90 (socioeconomic), .85 (spouse-partner), .90 (family-friends), and .82 (health and functioning). In a study by Choi [17], Cronbach’s α was .93, while in the current study, it was .97, with subdomain values of .90, .89, .88, .91, and .87, respectively.

#### Postpartum depression

PPD was assessed using the Edinburgh Postnatal Depression Scale (EPDS), developed by Cox et al. [24] and translated by Kim [25], which was used with permission. The EPDS consists of 10 items evaluating depressive mood, anxiety, and suicidal ideation experienced over the past week. Each item is rated on a 4-point Likert scale (0–3), yielding a total score between 0 and 30, with higher scores indicating more severe depression. Items with positive phrasing (items 1, 2, and 4) are scored directly, while the remaining items are reverse scored.

A cutoff score of 10 was used to classify participants into nondepressed (<10) and depressed (≥10) groups [25]. Cronbach’s α was reported as .87 at development, .83 in Kim’s study [26], and .89 in the present study.

#### Convergence of femininity-maternity

Convergence of maternity‐femininity refers to the process by which postpartum mothers maintain their feminine identity while adapting to their maternal role, thereby establishing a satisfying sense of motherhood. This study used an instrument developed by Lee and Cho [12], after permission. The instrument comprises 32 items measuring three subfactors of maternal adaptation patterns (self-concept, role-function, and interdependence modes) across eight factors: coexistence of femininity and maternity (three items), self-maturity (three items), body image (three items), establishment of the maternal role (three items), performance of maternal duties (seven items), adjustment of daily life (three items), outcomes of mother-infant interaction (seven items), and formation of a support system (three items). Items are rated on a 5-point Likert scale (1 not at all, to 5 very much), with total scores ranging from 32 to 160; higher scores indicate greater convergence of maternity‐femininity. The original reliability was Cronbach’s α=.91, and in this study, it was .94.

#### Marital intimacy

Marital intimacy was assessed using 15 items from the intimacy domain—one of three components (passion, intimacy, and commitment)—of Sternberg’s Triangular Theory of Love Scale [27]. These items were translated by Kwon [28] and later revised by Park and Park [29] with permission. Each item is rated on a 5-point Likert scale (1 not at all, to 5 completely) yielding total scores between 15 and 75; higher scores indicate greater marital intimacy. In Park and Park’s study [29], Cronbach’s α was .83, while in the present study, it was .93.

#### General and obstetric characteristics

General characteristics included participants’ age, education level, employment status, monthly income, and postpartum care assistance. Obstetric characteristics included postpartum weeks, feeding method, delivery method, and obstetric history.

### Data collection

Data were collected from March 17 to 30, 2023, using convenience sampling from the obstetrics and gynecology outpatient clinics of three general hospitals in Changwon, Korea, as well as an online community (N Cafe).

In the hospitals, recruitment was conducted after obtaining permission from the nursing departments, and notices were posted on outpatient bulletin boards. Participants who understood the study’s purpose and met the selection criteria accessed a QR code provided in the notice to voluntarily complete the online self-administered questionnaire. Similarly, recruitment announcements outlining the study and eligibility criteria were posted on an online community for mothers, where participants could access the questionnaire via a provided link.

The survey took approximately 10 to 15 minutes to complete, and participants were informed that they could withdraw at any time without penalty. Upon completion, they received a mobile coupon (worth about X US dollars) delivered within 1 week.

### Data analysis

The collected data were analyzed using IBM SPSS Statistics for Windows, ver. 25.0 (IBM Corp., Armonk, NY, USA) and Hayes’ PROCESS Macro 4.2 [30]. General and obstetric characteristics of postpartum mothers were analyzed using frequency, percentage, mean, and standard deviation. QoL, PPD, convergence of maternity‐femininity, and marital intimacy were assessed using mean, standard deviation, minimum, and maximum values. Differences in these variables based on general and obstetric characteristics were examined using independent t-tests and one-way analysis of variance with Scheffé post hoc tests. Pearson correlation coefficients were calculated to explore relationships among PPD, convergence of maternity‐femininity, marital intimacy, and QoL. To assess the mediating effects of convergence of maternity‐femininity and marital intimacy on the relationship between PPD and QoL, Hayes’ PROCESS Macro Model 4 was applied with a 95% bias‐corrected bootstrap confidence interval (CI) based on 10,000 bootstrap samples.

## Results

### General and obstetric characteristics

The average age of the participants was 32.52±3.20 years, with 169 (85.8%) in their 30s. Among them, 144 (73.1%) were university graduates, 108 (54.8%) were full-time homemakers, and 165 (83.8%) reported a monthly family income of at least KRW 3.1 million.

Regarding obstetric characteristics, 134 participants (68.0%) had vaginal deliveries, and 167 (84.8%) were primigravid. The mean postpartum period was 4.28±1.04 weeks, with 149 (75.6%) being 4 to 6 weeks postpartum. Breastfeeding was the most common feeding method, reported by 88 participants (44.7%) (Table 1).

### Postpartum quality of life, postpartum depression, convergence of femininity-maternity, and marital intimacy

Participants’ QoL averaged 17.78±3.83, indicating a somewhat low level. The average PPD score was 12.06±6.79, exceeding the cutoff of 10 and suggesting a relatively high level of depressive symptoms. Marital intimacy averaged 55.95±10.40, indicating a moderate level, while the convergence of maternity‐femininity score averaged 118.84±19.85. However, interpretation of this score remains tentative due to limited previous research (Table 2).

### Differences in postpartum quality of life, postpartum depression, convergence of femininity-maternity, and marital intimacy based on general and obstetric characteristics

No significant differences in QoL were observed based on general or obstetric characteristics. However, PPD varied significantly by postpartum week (t=16.64, *p*=.020), with mothers in the early postpartum period reporting higher levels of depression than those in later weeks. Additionally, marital intimacy differed significantly by obstetric history (t=2.40, *p*=.022), with primigravid participants reporting higher intimacy than multiparous participants.

### Relationships among quality of life, postpartum depression, convergence of femininity-maternity, and marital intimacy in postpartum mothers

QoL was negatively correlated with PPD (r=–.70, p<.001) and positively correlated with both convergence of maternity‐femininity (r=.79, *p*<.001) and marital intimacy (r=.70, *p*<.001) (Table 3).

### Mediating effects of convergence of femininity-maternity and marital intimacy on the relationship between postpartum depression and quality of life

Before conducting the mediation analysis, the assumptions for regression analysis were verified. Skewness values ranged from –0.70 to –0.03 and kurtosis values from –0.93 to 0.52, meeting normality criteria. Tolerance values ranged from 0.31 to 1.00 (exceeding 0.1), and variance inflation factors ranged from 1.00 to 3.19 (below 10), indicating no multicollinearity issues. The Durbin-Watson statistic was 2.01, close to 2.00, indicating no concerns with autocorrelation.

**Figure 1.**
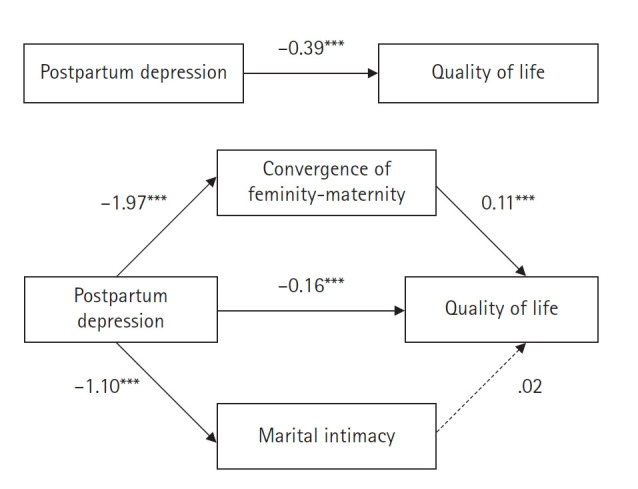
Mediating effects of convergence of femininity-maternity and marital intimacy in the relationship between postpartum depression and quality of life in postpartum mothers. ^**^p<.01, ^***^p<.001.

A double mediation model was analyzed using Hayes’ PROCESS Macro Model 4 [30] to examine the mediating effects of convergence of maternity‐femininity and marital intimacy on the relationship between PPD and QoL among postpartum mothers (Table 4, Figure 1). Bootstrapping with 10,000 samples and a 95% CI was used to test the significance of the mediating effects (Table 5).

The analysis revealed that PPD significantly negatively affected both convergence of maternity‐femininity (B=–1.97, *p*<.001) and marital intimacy (B=–1.10, *p*<.001). In turn, both PPD (B=–0.16, *p*<.001) and convergence of maternity‐femininity (B=0.11, *p*<.001) significantly influenced QoL, whereas marital intimacy did not (B=0.02, *p*=.543) (Table 4). The total effect of PPD on QoL (B=–0.39, *p*<.001) decreased to a direct effect of (B=0.16 (*p*<.001) when the mediators were included (Figure 1). Bootstrapping revealed that the indirect effect via convergence of maternity‐femininity was –0.22 (95% CI, –0.28 to –0.15), which was significant because the CI did not include 0. In contrast, the indirect effect via marital intimacy was –0.02 (95% CI, –0.09 to 0.06), which was not significant. Thus, convergence of maternity‐femininity demonstrated a partial mediating effect on the relationship between PPD and QoL, whereas marital intimacy did not function as a mediator (Table 5).

According to Shrout and Bolger [31], the strength of a mediating effect can be determined by the proportion of the total effect explained by the indirect effect. The ratio of the indirect effect to the total effect revealed that convergence of maternity‐femininity partially mediated the relationship between PPD and QoL, accounting for 56.0% of the total effect.

## Discussion

This study found that convergence of maternity‐femininity partially mediated the relationship between PPD and QoL among postpartum mothers, whereas marital intimacy—although affected by PPD—did not serve as a significant mediator. This suggests that while PPD directly impacts QoL, it can also indirectly affect it through convergence of femininity-maternity. Although direct comparisons with previous studies are limited due to the paucity of research on convergence of maternity‐femininity in the postpartum period, these results align with prior findings. For example, earlier research on mothers in the early postpartum period has shown that PPD negatively affects maternal role adaptation [17], which in turn positively influences QoL [18]. Thus, lower levels of PPD appear to be associated with better maternal role adaptation and enhanced confidence in one’s femininity, ultimately improving QoL.

To improve postpartum mothers’ QoL, nursing interventions should aim both to reduce PPD and to promote convergence of maternity‐femininity. Because convergence of maternity‐femininity is influenced by PPD, comprehensive assessments and targeted interventions are essential when establishing nursing strategies. Building on the findings of this study, it is necessary to develop and evaluate programs specifically designed to support convergence of maternity‐femininity in postpartum mothers.

Marital intimacy was not identified as a significant mediator between PPD and QoL. Although this contrasts with previous studies that reported marital intimacy as influencing QoL [32], it does not imply that marital intimacy should be disregarded. Future research should further explore the role of marital intimacy in relation to other variables affecting QoL.

In this study, participants’ QoL averaged 17.77±3.83, which is lower than the 19.78 reported for mothers 1 to 3 weeks postpartum using the same instrument [4]. This difference suggests that the coronavirus disease 2019 (COVID-19) pandemic—with its associated fear of infection, reduced social support, and limited external contact—may have adversely affected QoL. Comparative studies across various regions after the pandemic subsides are warranted.

The health and functioning subdomain emerged as the lowest aspect of QoL. This finding aligns with previous research identifying it as the weakest domain [4]. International studies have similarly found this domain to rank lowest during both the first and third weeks postpartum [20], and both domestic and international studies have consistently highlighted health and functioning as the poorest aspect of QoL [22]. Therefore, providing basic health counseling on nutrition, exercise, and sleep for mothers and their newborns is essential.

Overall QoL did not differ significantly based on general or obstetric characteristics. However, in the spouse-partner relationship domain, primiparous mothers scored significantly higher than multiparous mothers. One interpretation is that multiparous mothers, already burdened by responsibilities and parenting stress related to older children, must allocate additional time and energy after delivery to care for both newborns and older children. This may leave them with less time to nurture their spousal relationship, reducing the quality of their spousal relationship.

The participants’ PPD averaged 12.06±6.79 on a 30-point scale—exceeding the cutoff of 10. In contrast, previous studies using the same instrument reported PPD scores of 8.65±0.43 for mothers 2 to 6 weeks postpartum [33] and 8.00±4.37 for mothers 1 to 3 weeks postpartum [4]. These findings suggest that PPD levels in this study were higher, possibly linked to the COVID-19 pandemic. Infection prevention measures restricted visits from family and acquaintances, reducing support for postpartum mothers, while concerns about infection for themselves, their infants, and their families likely exacerbated PPD symptoms [34]. To address this, it is crucial to provide education on infection prevention for mothers and their close contacts, along with mental health support programs—potentially delivered via remote modalities—when necessary.

PPD was significantly higher during the early postpartum period (average, 13.77±5.16) compared to the late postpartum period (average, 11.53±7.19). This aligns with previous research using the same instrument, which reported early postpartum scores of 6.72±4.30 and late postpartum scores of 6.12±3.33 [21]. These results underscore the importance of considering time elapsed since childbirth when assessing PPD levels [35]. Because mothers in the early postpartum period (1–3 weeks) are more vulnerable to PPD, targeted nursing interventions during outpatient visits are crucial. Clinical nurses should prioritize early identification of PPD symptoms, develop tailored treatment plans, and provide resources such as emotional support, psychotherapy, social connection, and parenting assistance to alleviate PPD and improve QoL.

Currently, health centers in various regions identify high-risk mothers through PPD screening and refer them to Mental Health Welfare Centers or medical institutions for further management, such as psychiatric consultations. However, the availability of specialized counseling centers remains limited and unevenly distributed across regions [36]. Expanding access to these services nationwide is essential for effective PPD management.

This study found that the convergence of maternity‐femininity score was 118.40±20.74 out of a range of 32-160. Due to limited previous research, it is challenging to compare the degree of convergence. Future studies focusing on postpartum mothers are needed to further explore this concept. Nonetheless, these findings provide foundational data for developing nursing interventions to enhance convergence of maternity‐femininity.

The marital intimacy score was 55.95±10.40 out of 75, with an average item score of 3.73±0.69. A previous study using the same instrument reported an average item score of 3.52±0.28, indicating a similar level of marital intimacy. However, due to limited research on marital intimacy among postpartum mothers, direct comparisons remain difficult, and further investigation is needed. Additionally, since the instrument used was designed for married women of all ages, it is necessary to develop and validate customized scales specifically tailored for postpartum mothers.

This study is significant because it investigated the relationships among PPD, marital intimacy, convergence of maternity‐femininity, and QoL in postpartum mothers 2 to 6 weeks after childbirth, while also analyzing the mediating effects of convergence of maternity‐femininity and marital intimacy. The findings emphasize the importance of not only preventing PPD as a strategy for improving QoL but also promoting convergence of maternity‐femininity through targeted educational interventions. Moreover, since family support plays a key role in strengthening convergence of maternity‐femininity, the results may serve as valuable educational material. However, because convenience sampling was used from a general hospital in a mid-sized city and an online community, the generalizability of the findings is limited. Future research should include postpartum mothers from a broader range of regions.

In conclusion, improving postpartum mothers’ QoL requires interventions that both reduce PPD and promote convergence of maternity‐femininity. Because femininity-maternity convergence is influenced by PPD, interventions to improve it should be designed based on individual PPD assessments. Postpartum mothers should have access to regular psychological counseling that not only addresses PPD but also supports maintaining their feminine identity while transitioning into motherhood. Physical well-being also plays a crucial role—studies have shown that postnatal exercise programs can improve body image, thereby enhancing convergence of maternity‐femininity [12] and potentially reducing PPD [37]. Consequently, appropriate exercise programs tailored to the circumstances of postpartum mothers should be offered. Most importantly, mothers need support that allows them time for self-care. The Korean government has taken steps in this direction through the 2023 Public Postnatal Care Service for Mothers and Newborns, which includes postpartum doula services [38]. To maximize the impact of these programs, increased promotion and support from both government and local authorities is essential. Such a multifaceted approach can significantly enhance the QoL of postpartum mothers.

## Figures and Tables

**Table 1.** General and obstetric characteristics (N=197)

Characteristic	Category	n (%) or Mean±SD
Age (year)		32.52±3.20
	20–29	28 (14.2)
	≥30	169 (85.8)
Postpartum week		4.28±1.04
	Early postpartum period (2–3 weeks)	48 (24.4)
	Late postpartum period (4–6 weeks)	149 (75.6)
Education	≤High school graduate	10 (5.1)
	Community college graduate	43 (21.8)
	≥University graduate	144 (73.1)
Employed	Yes	108 (54.8)
	No	89 (45.2)
Monthly income (Korean won)^†^	≤3 million	32 (16.2)
	>3 million	165 (83.8)
Feeding method	Breastfeeding	88 (44.7)
	Formula feeding	46 (23.3)
	Mixed feeding	63 (32.0)
Delivery method	Vaginal delivery	134 (68.0)
	Cesarean section	63 (32.0)
Obstetric history	Primigravida	167 (84.8)
	Multigravida	30 (15.2)

† One million Korean won is approximately 696.65 US dollars.

**Table 2.** Postpartum quality of life (QoL), postpartum depression, marital intimacy, and convergence of femininity-maternity (N=197)

Variable	Mean±SD	Possible range	Data range
Postpartum QoL	17.78±3.83	0–30	7.25–26.60
Psychological	18.39±4.37	0–30	6.81–29.06
Socioeconomic	17.78±4.15	0–30	8.25–26.75
Relation/spouse-partner	18.68±4.67	0–30	5.00–30.00
Relation/family-friends	17.86±4.15	0–30	5.95–28.15
Health and functioning	16.39±3.98	0–30	5.31–27.06
Postpartum depression	12.06±6.79	0–30	0–28
Non-depressed group (score <10)^†^	4.14±2.76		0–9
Depressed group (score ≥10)^‡^	16.45±3.69		10–28
Convergence of femininity-maternity	118.84±19.85	32–160	60–156
Self-concept mode	33.76±5.67	9–45	17–45
Role-function mode	47.20±8.84	13–65	23–64
Interdependence mode	38.44±7.61	10–50	18–50
Marital intimacy	55.95±10.40	15–75	22–75

† n=70 (35.5%).

‡ n=127 (64.5%).

**Table 3.** Correlations between quality of life (QoL), postpartum depression, convergence of femininity-maternity, and marital intimacy in postpartum mothers (N=197)

Variable	r (*p*)			
	Postpartum QoL	Postpartum depression	Convergence of femininity-maternity	Marital intimacy
Postpartum QoL	1			
Postpartum depression	–.70 (<.001)	1		
Convergence of femininity-maternity	.79 (<.001)	–.67 (<.001)	1	
Marital intimacy	–.70 (<.001)	–.72 (<.001)	.79 (<.001)	1

**Table 4.** The mediating effect of CFM and MI in the relationship between PPD and QoL (N=197)

Variable	B	SE	95% CI	*p*-value
PPD → CFM	–1.97	0.15	–2.27 to –1.66	<.001
PPD → MI	–1.10	0.08	–1.25 to –0.95	<.001
PPD → QoL	–0.16	0.03	–0.23 to –0.09	<.001
CFM → QoL	0.11	0.01	0.08 to 0.14	<.001
MI → QoL	0.02	0.03	–0.04 to 0.07	.543

CFM, Convergence of femininity-maternity; MI, marital intimacy; PPD, postpartum depression; QoL, quality of life.

**Table 5.** Verification of the mediating effect of CFM and MI (N=197)

Variable	Direct effect				Indirect effect			
	B	Boot SE	95% CI	*p*-value	B	Boot SE	95% CI	*p*-value
PPD → QoL	–0.16	0.03	–0.23 to -0.09	<.001				
PPD → CFM → QoL					–0.22	0.03	–0.28 to -0.15	<.001
PPD → MI → QoL					–0.02	0.04	–0.09 to 0.06	>.543
Total					–0.23	0.03	–0.30 to –0.17	<.001

CI, Confidence interval; CFM, convergence of femininity-maternity; MI, marital intimacy; PPD, postpartum depression; QoL, quality of life.
